# Identification of Intercellular Signaling Changes Across Conditions and Their Influence on Intracellular Signaling Response From Multiple Single-Cell Datasets

**DOI:** 10.3389/fgene.2021.751158

**Published:** 2021-11-11

**Authors:** Mengqian Hao, Xiufen Zou, Suoqin Jin

**Affiliations:** ^1^ School of Mathematics and Statistics, Wuhan University, Wuhan, China; ^2^ Hubei Key Laboratory of Computational Science, Wuhan University, Wuhan, China

**Keywords:** scRNA-seq data, intercellular communication, intracellular signaling, multiscale signaling network, dysregulated signaling, comparison analysis

## Abstract

Identification of intercellular signaling changes across multiple single-cell RNA-sequencing (scRNA-seq) datasets as well as how intercellular communications affect intracellular transcription factors (TFs) to regulate target genes is crucial in understanding how distinct cell states respond to evolution, perturbations, and diseases. Here, we first generalized our previously developed tool CellChat, enabling flexible comparison analysis of cell–cell communication networks across any number of scRNA-seq datasets from interrelated biological conditions. This greatly facilitates the ready detection of signaling changes of cell–cell communication in response to any biological perturbations. We then investigated how intercellular communications affect intracellular signaling response by inferring a multiscale signaling network which bridges the intercellular communications at the population level and the cell state–specific intracellular signaling network at the molecular level. The latter is constructed by integrating receptor-TF interactions collected from public databases and TF-target gene regulations inferred from a network-regularized regression model. By applying our approaches to three scRNA-seq datasets from skin development, spinal cord injury, and COVID-19, we demonstrated the capability of our approaches in identifying the predominant signaling changes across conditions and the critical signaling mechanisms regulating target gene expression. Together, our work will facilitate the identification of both intercellular and intracellular dysregulated signaling mechanisms responsible for biological perturbations in diverse tissues.

## Introduction

Cell–cell communication means that one cell sends a message to another cell through a medium to initiate cellular response of the target cell. The communication between cells plays a vital role in the development, physicology, and pathology of muticellular organisms. In this process, cells can communicate with and respond to neighboring or distant cells through ligand-receptor interactions by utilizing biochemical molecules, such as cytokines and growth factors. Single-cell RNA-sequencing (scRNA-seq), which measures expression levels of a large number of genes across many cell types at a single-cell resolution, provides a great opportunity to study the cell–cell communication between interacting cells and the signaling response governed by intracellular gene regulatory networks (GRNs) (Almet, et al., 2021; Shao, et al., 2020). Moreover, identification of signaling changes across conditions is important for understanding how distinct cell states respond to evolution, perturbations, and diseases (Armingol, et al., 2021).

Although a number of computational methods have been recently developed to infer cell–cell communication by integrating scRNA-seq data with a prior ligand–receptor interaction database, most of these methods only focus on the intercellular communications in one biological condition (Almet, et al., 2021; Armingol, et al., 2021), lacking the capability of identifying signaling changes across conditions. We have recently developed a computational tool CellChat (Jin, et al., 2021) to identify dysregulated interactions by comparing cell–cell communication networks across conditions. However, CellChat focuses primarily on the comparison analysis between two datasets from two interrelated biological conditions. Other methods, including iTalk (Wang, et al., 2019) and Connectome (Raredon, et al., 2021), have also been developed recently to perform comparison analysis. With the increasing number of scNRA-seq datasets collected from multiple conditions, time points, and disease states, easy-to-use tools that can seamlessly identify signaling changes across any biological conditions from multiple scRNA-seq datasets are highly needed.

Understanding how cell–cell communication affects the gene expression of target cells via transcription factors (TFs) is crucial to understand how target cells respond to extracellular signals and eventually the functional role of cell–cell communication. However, there are only rudimentary efforts to link cell–cell communication to downstream response *via* GRNs (Browaeys, et al., 2020; Cheng, et al., 2021; Hu, et al., 2020; Sha, et al., 2020), such as NicheNet, scMLnet, and CytoTalk. NicheNet and scMLnet build GRNs by directly curating the interactions among ligands, receptors, TFs, and target genes from public databases, while CytoTalk infers GRN by calculating the mutual information between all pairs of genes without discriminating TFs from target genes. Constructing a multiscale signaling network, which links data-driven intercellular communications with intracellular TF-target regulations, still remains challenging, preventing the better understanding of cell type–specific response to cell–cell communication.

To address these limitations, we first generalized our previously developed R package CellChat to enable the comparison analysis of any number of datasets from multiple conditions, allowing ready identification of signaling changes across conditions. In addition, we infer a multiscale signaling network which integrates the ligand–receptor interactions inferred from CellChat, the receptor-TF interactions from public databases, and the TF- gene regulations from a mathematical optimization model taking into account the prior network information from public databases. Of note, we build cell type–specific networks from the integrated network by identifying enriched TFs and target genes based on the differential expression analysis. Therefore, our multiscale framework provides a clear understanding of how the upstream of the signaling pathway in cell–cell communication regulates the downstream target genes in a sequential way. We apply our approaches to three scRNA-seq datasets from mouse skin embryonic development, mouse spinal cord injury, and human COVID-19 infection. Applications not only demonstrate the capability of our methods but also provide novel insights into signaling mechanisms driving phenotype transitions.

## Results

### Overview of Identifying Intercellular Signaling Changes Across Conditions and Their Link to Intracellular Signaling Response From Multiple scRNA-Seq Datasets

**FIGURE 1:**
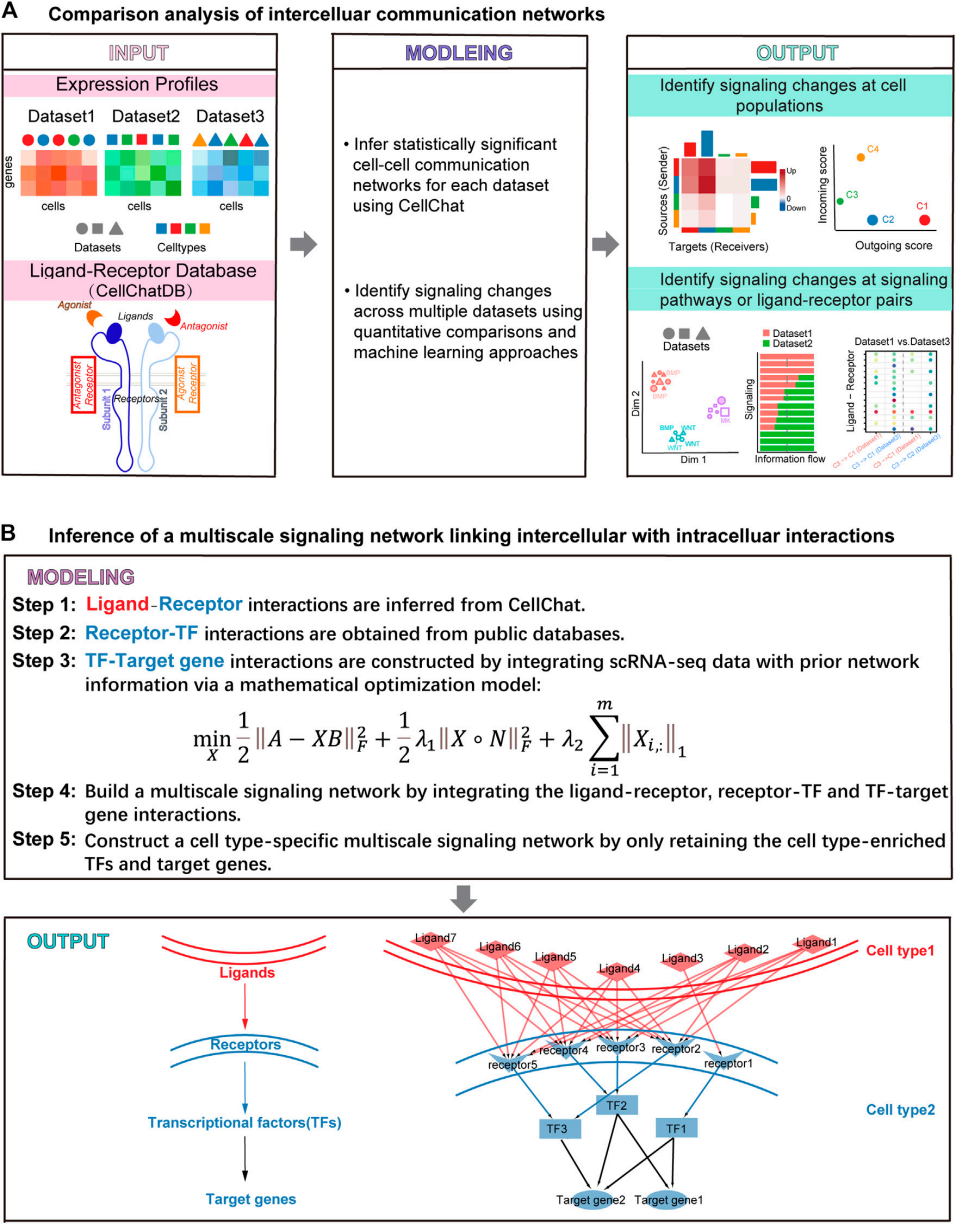
Overview of identifying intercellular signaling changes across conditions and inferring the multiscale signaling network from multiple scRNA-seq datasets. **(A)** The generalized CellChat identifies intercellular signaling changes across conditions from multiple scRNA-seq datasets. CellChat requires the users to provide multiple datasets with cell type labels as input, where the cell types of different data sets may not be exactly the same. CellChat identifies biologically significant signaling pathways for each dataset separately and then performs comparative analysis across multiple datasets in a systematic and quantitative manner. CellChat identifies signaling changes across multiple datasets in terms of cell types and signaling pathways or ligand receptor pairs. Different plots are provided to allow ready comparison analysis. **(B)** Multiscale signaling network is inferred to link intercellular communication to intracellular signaling, which integrates the ligand–receptor interactions, receptor-TFs interactions, and TFs-target gene interactions.

We first generalized our previously developed tool CellChat together with the R package, providing a more coherent and easy-to-use way to perform comparison analysis of cell–cell communication across conditions from any number of scRNA-seq datasets. Cellchat requires users to provide a scRNA-seq dataset (gene expression data across cells) with cell type labels as the input (Figure 1A). After receiving the input information, CellChat infers statistically and biologically significant cell–cell communication networks for each dataset. Compared to the original CellChat that was limited to the comparison analysis of only two datasets, the updated CellChat generalizes many existing functions, which enables systematical comparison analysis of intercellular communications across any number of scRNA-seq datasets. Of note, cell type compositions in different datasets do not need to be exactly the same. Moreover, by introducing a merged CellChat object from a list of CellChat objects, the updated CellChat allows the comparison analysis of cell–cell communication networks across all input datasets in a coherent and flexible fashion. Specifically, CellChat can identify the changes of the dominant sender and receiver in cell groups by comparing any two datasets using network centrality metrics such as out-degree and in-degree. CellChat can also identify the predominantly altered signaling pathways and ligand–receptor pairs by comparing the inferred communication probabilities and projecting the inferred cell–cell communication networks onto a shared low-dimensional space for any number of datasets. CellChat displays the results of comparative analysis of multiple datasets in a variety of intuitive visualization methods, such as scatter plots, heatmaps, bar plots, and bubble plots (Figure 1A).

We then build a multiscale signaling network by integrating the cell–cell communication between interacting cells (i.e., intercellular communication) with the downstream signaling inside target cells (i.e., intracellular signaling). The intercellular communication is given by CellChat while the intracellular signaling is inferred by constructing a gene–gene network linking receptors, TFs, and target genes. Specifically, the construction of a multiscale signaling network includes five steps. Step 1, the intercellular communication mediated by ligand–receptor interactions is obtained by CellChat. Step 2, the receptor-TF subnetwork is collected from a comprehensive database OmniPath (Türei, et al., 2016; Türei et al., 2021). Step 3, the TF-target gene subnetwork is inferred by integrating the TF activity data, the gene expression data, and the prior network information from the OmniPath database *via* a network-regularized regression model (MATERIALS AND METHODS). The TF activity is estimated based on their target gene expression in the scRNA-seq data using the widely used method DoRothEA (Garcia-Alonso, et al., 2019). Step 4, the multiscale signaling network is constructed by integrating the intercellular communication network, the receptor-TF network, and TF-target gene network, which links intercellular communications with intracellular signaling response. Step 5, the cell type–specific multiscale signaling network is finally constructed by only retaining the cell type–enriched TFs and target genes based on differential expression analysis (Figure 1B).

Together, our new approaches will advance our understanding of signaling mechanisms by identifying signaling changes that potentially drive phenotype transitions and by constructing multiscale signaling networks that imply how intercellular communications affect intracellular TFs to regulate target gene expression.

### Comparison Analysis Predicts WNT Signaling as a Predominant Signaling Change During Mouse Embryonic Skin Development

To demonstrate the capability of our approaches in capturing predominant signaling changes across multiple time points, we first applied our generalized CellChat to our previously published mouse skin scRNA-seq datasets, which described epidermal development at three embryonic stages: E14.5, E16.5, and E18.5 (newborn) (Lin, et al., 2020). Unsupervised clustering identified five interfollicular epidermis (IFE) cell states: two basal cell states (IFE-B.1 and IFE-B.2), two transition cell states (IFE-T.1 and IFE-T.2), differentiated cells (IFE-D), and terminally differentiated cells (IFE-TD) (Lin, et al., 2020) (Supplementary Figure S1A).

**FIGURE 2:**
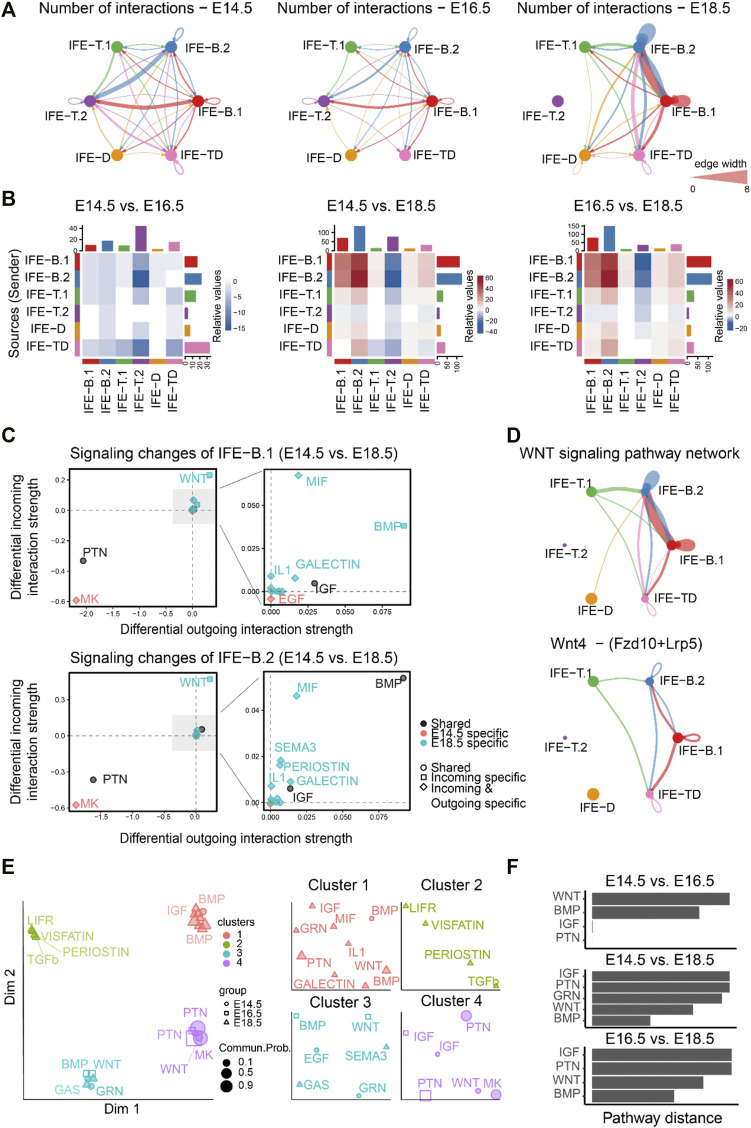
Comparison analysis predicts WNT signaling as a predominant signaling change during mouse embryonic skin development. **(A)** The comparison of the total number of interactions among different cell populations between E14.5, E16.5, and E18.5. Edge width is proportional to the number of interactions, which assess how many ligand–receptor pairs contributing to the communication between two interacting cell populations. **(B)** Heatmap showing the differential number of interactions between E14.5, E16.5, and E18.5. In the color bar, red (or blue) represents increased (or decreased) signaling in the second dataset compared to the first one. **(C)** Identifying the specific signaling changes of IFE-B.1 and IFE-B.2 from E14.5 to E18.5. **(D)** The inferred WNT signaling pathway network and Wnt4 - (Fzd10 + Lrp5) signaling network at E18.5. **(E)** Projection and classification of signaling networks from E14.5, E16.5, and E18.5 onto a two-dimensional space based on the network similarity. Different shapes represent signaling networks from different developmental stages. **(F)** Computing the pathway distance of the signaling network from E14.5, E16.5, and E18.5 based on their Euclidean distance in the shared two-dimensional space.

To study how the cell–cell communication changes across different stages during mouse embryonic development, we first compared the number of inferred interactions among different cell populations among E14.5, E16.5, and E18.5 (Figures 2A,B). We observed slightly decreased cell–cell communication at E16.5 compared to E14.5, but significantly dynamic changes at E18.5 compared to both E14.5 and E16.5, suggesting dramatic signaling changes from E16.5 to E18.5 at the later embryonic stages. In particular, both outgoing and incoming signaling associated with IFE-B.1 and IFE-B.2 was predominantly increased at E18.5 compared to both E14.5 and E16.5. Surprisingly, our results showed that IFE-T.2 does not have any communication with any cell populations, which is likely due to the very few number of cells in IFET.2 at E18.5 (Figure 2A and Supplementary Figure S1A).

Moreover, to identify the signaling pathways contributing to the dramatic signaling changes of IFE-B.1 and IFE-B.2, we calculated the differential outgoing and incoming interaction strength of each signaling pathway between E14.5 and E18.5. For both IFE-B.1 and IFE-B.2, we observed WNT signaling as the most predominantly increased signaling at E18.5 compared to E14.5, as reflected by the largest differential outgoing and incoming interaction strength compared to other signaling pathways (Figure 2C), which was in agreement with the previous finding. In addition to WNT signaling, we also observed other increased signaling changes for both outgoing and incoming signaling including BMP, MIF, GALECTIN, and IL1, and decreased signaling including MK and PTN (Figure 2C). Attractively, our previous study experimentally showed that WNT-secreting stem cells play a central role in IFE self-renewal during homeostasis, which can inhibit the expansion of epidermal stem cells and the appearance of abnormal stem cell states (Lin, et al., 2020), in particular Wnt4 signaling. Indeed, we calculated the contribution of each ligand–receptor pair to the WNT signaling pathway and observed that Wnt4 - (Fzd10 + Lrp5) makes a relatively large contribution (Supplementary Figure S1B). By examining the gene expression levels of the ligand Wnt4 and its receptor Fzd10 and coreceptor Lrp5, IFE-B.1 and IFE-B.2 exhibited relatively high expression (Supplementary Figure S1C). Consistent with these observations, the inferred cell–cell communication networks of the WNT signaling pathway and the ligand–receptor pair Wnt4 - (Fzd10 + Lrp5) showed that IFE-B.1 and IFE-B.2 are the dominant signaling sources and targets at E18.5. In addition, IFE-T.1 and IFE-TD emerge as the signaling source and target, respectively, helping drive the complexity of WNT signaling.

We next investigated how the cell–cell communication architecture changes by projecting the inferred cell–cell communication networks from the three development stages onto a shared two-dimensional space based on whether they have similar signaling sources and targets (MATERIALS AND METHODS). This analysis classified all significant signaling pathways into four groups. Interestingly, the shared signaling pathways from two development stages were classified into different groups, such as WNT, BMP, and IGF (Figures 2E,F), suggesting that these pathways changed their cell–cell communication architecture during embryonic development.

Together, comparison analyses of the inferred cell–cell communication networks across the three embryonic development stages suggest the dramatic signaling changes at the later stages and revealed WNT signaling as a predominant signaling change during mouse embryonic skin development.

### Comparison Analysis Reveals Myeloid Cells-Mediated Signaling Mechanisms and Pinpoints the Key Time Point of Signaling Changes in Response to Mouse Spinal Cord Injury

Next, we demonstrate how our generalized CellChat can be applied in studying temporal changes of intercellular communications over four time points using a recently published mouse spinal cord injury sc-RNAseq dataset (Milich, et al., 2021). This dataset describes the wound healing process that occurs after spinal cord injury over four time points, including the uninjured and injured spinal cord at 1, 3, and 7 days postinjury (dpi). 66,176 cells were classified into 15 distinct cell groups: microglia, astrocytes, monocytes, macrophages, neutrophils, div-myeloid cells, dendritic cells, lymphocytes, oligodendrocytes (OLs), OPCs, neurons, fibroblasts, pericytes, ependymal cells, and endothelial cells.

**FIGURE 3:**
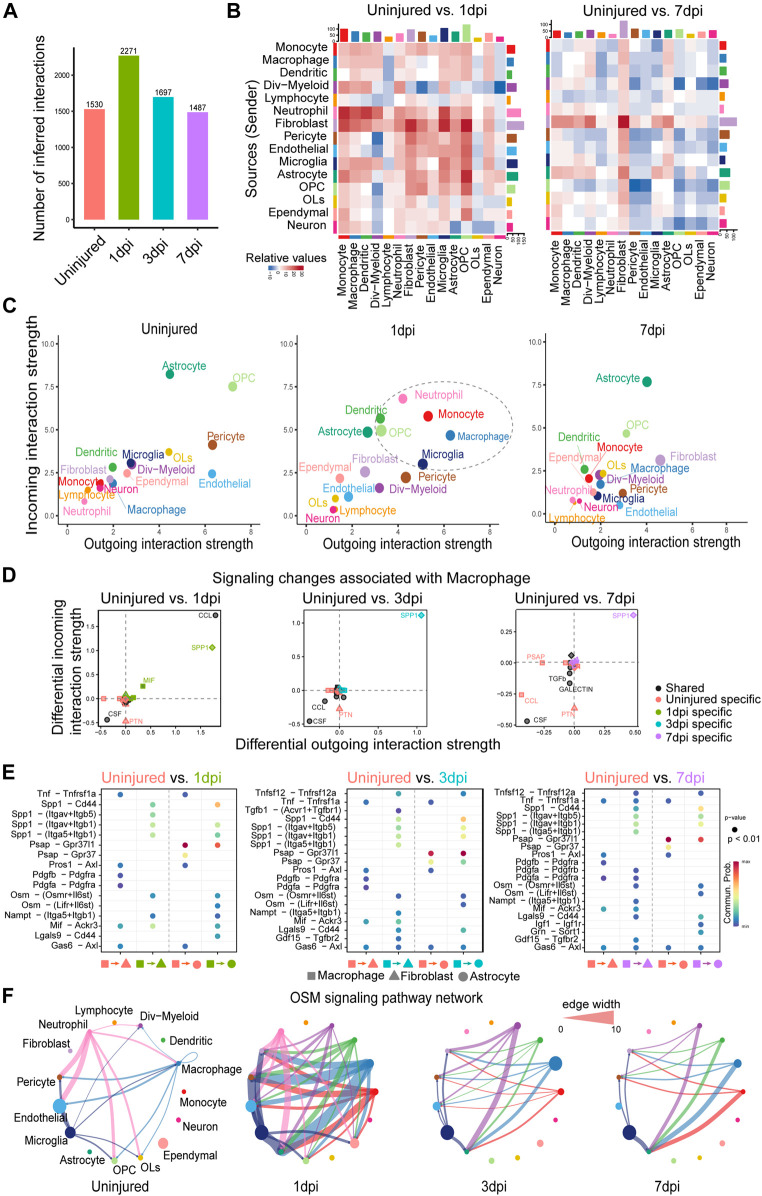
Comparison analysis reveals myeloid cell–mediated signaling mechanisms and pinpoints the key time point of signaling changes in response to mouse spinal cord injury. **(A)** Comparison of the total number of interactions of the inferred cell–cell communication networks from uninjured, 1, 3, and 7dpi. **(B)** Heatmaps of the differential number of interactions between uninjured and 1dpi as well as uninjured and 7dpi, showing the outgoing and incoming signaling change of each cell group in a greater detail (The top colored bar plot represents the sum of each column of values displayed in the heatmap (incoming signaling). The right colored bar plot represents the sum of each row of values (outgoing signaling). **(C)** Scatter plots comparing the outgoing and incoming interaction strength in the 2D space among uninjured, 1dpi, and 7dpi. **(D)** Identifying signaling changes associated with macrophages by comparing uninjured with 1, 3, and 7dpi, respectively. **(E)** Identification of dysfunctional signaling by comparing the communication probabilities mediated by ligand–receptor pairs from macrophages to astrocytes and fibroblasts. **(F)** Circle plots displaying the inferred network of the OSM signaling pathway at uninjured, 1, 3, and 7dpi. Edge width is proportional to the inferred communication probabilities.

We first compared the total number of interactions (i.e., the number of ligand–receptor pairs contributing to communication between any two interacting cell groups) that were inferred by CellChat over spinal cord injury. We found that the number of cell–cell communication was significantly increased at 1dpi after spinal cord injury, but afterwards decreased to its basal level by 7dpi (Figure 3A), suggesting that 1dpi was a critical time point where cell–cell communication between different cell types was significantly enhanced. To find out the interaction between which cell groups was significantly changed, we computed the differential number of interactions for both outgoing and incoming signaling of pairwise cell groups between any pair of two time points. We observed that the number of interactions between cell groups at 1dpi was mostly increased compared to the uninjured, while cell–cell communication at 3dpi and 7dpi exhibited a dynamic change with both increased and decreased interactions (Figure 3B). Interestingly, both outgoing signaling and incoming signaling of fibroblasts and astrocytes were consistently enhanced at 1dpi, 3dpi, and 7dpi compared to the uninjured, consistent with the known important role of fibroblasts in tissue repair (Plikus, et al., 2021) (Figure 3B and Supplementary Figure S2A).

In addition, we studied how the major signaling sources and targets changed after injury. Compared to the uninjured tissue, we found that both the outgoing and incoming interaction strength of several myeloid cell populations, including the macrophage, monocyte, neutrophil, microglia, and dendritic cells, were significantly increased at 1dpi, and the outgoing and incoming interaction strength of fibroblasts was increased at 3dpi and then, further enhanced at 7dpi (Figure 3C and Supplementary Figure S2B). These results agreed well with the previous findings: 1) At 1dpi, peripheral myeloid cells, mainly neutrophils and monocytes, migrate to the injury site and then enhance the innate immune response initiated by the microglia (Milich, et al., 2019); 2) Fibrosis is initiated at 3dpi and the number of fibroblasts reaches its peak at 7dpi (Zhu, et al., 2015). These two findings suggest the potential role of myeloid cells in initiating fibrosis after spinal cord injury.

To identify myeloid cell–mediated mechanisms of fibrosis, we examined signaling changes associated with macrophages by comparing its outgoing and incoming interaction strength of each signaling pathway at 1, 3, and 7dpi with the uninjured tissue. SPP1 signaling consistently exhibited the predominantly increased outgoing and incoming interaction strength at 1, 3, and 7dpi compared to the uninjured (Figure 3D), suggesting the important role of SPP1 signaling after spinal cord injury. This is consistent with the known neuroprotective roles of SPP1 and the worse histopathology and behavioral recovery in SPP1-knockout mice after spinal cord injury (Milich, et al., 2021). In addition, CCL signaling was also clearly increased at 1dpi compared to the uninjured (Figure 3D), which agreed with the innate immune response initiated by myeloid cells at 1dpi (Milich, et al., 2019). Furthermore, comparing the communication probabilities mediated by ligand–receptor pairs from macrophages to fibroblasts and astrocytes, we identified ligand-receptor pairs that were only enriched at 1, 3, and 7dpi, including SPP1 signaling such as Spp1 - (Itgav + Itgb5) and Spp1 - (Itga5+Itgb1) and OSM signaling such as Osm - (Osmr + Il6st) (Figure 3E and Supplementary Figure S2C). Consistent with our prediction, the previous study showed that OSM is a common mechanism by which fibroblasts and astrocytes are preferentially activated by monocyte/macrophage subtypes after spinal cord injury (Milich, et al., 2021). By examining the inferred cell–cell communication network at each time point, we found that OSM signaling was strongly activated with more signaling sources and a stronger interaction strength at 1dpi (Figure 3F). Compared to the uninjured tissue, other myeloid cells, including the monocyte, dendritic cells, and dividing myeloid cells (div-myeloid), emerged as new signaling sources, helping enhance the cell–cell communication driven by the macrophage. Notably, fibroblasts and astrocyte cells emerged as new signaling targets after injury, suggesting the myeloid cell–mediated signaling mechanisms of fibrosis. Taken together, our systematical comparison analysis pinpoints 1dpi as the key time point of signaling changes in response to spinal cord injury and reveals myeloid cell–mediated signaling mechanisms of fibrosis after mouse spinal cord injury.

### Comparison Analysis Identifies Crucial Signaling Changes Responsible for Disease Severity Related to COVID-19

Due to the ongoing pandemic caused by the new coronavirus (SARS-CoV-2), it is of great significance to investigate the level of cell-to-cell communication in patients with different severity of diseases related to COVID-19. We used scRNA-seq data from 19 patients with COVID-19 and five SARS-CoV-2-negative donors with no signs of the disease. This dataset includes five control cases, eight moderate cases, and eleven critical cases. In the control, moderate, and critical samples, each contains 2,982, 82,814, and 49,804 cells. We performed downsampling of the moderate and critical samples by randomly taking 20,000 cells from each sample without losing any cell population. This dataset comprises 20 cell populations, including ciliated-diff cells (differentiating ciliated), secretory-diff cells (differentiating secretory), ciliated cells, FOXN4+ cells, squamous cells, secretory cells, cytotoxic T lymphocytes (CTL), natural killer T cells (NKT), B lymphocytes (BC), plasmacytoid dendritic cells (pDC), monocyte-derived macrophages (moMa), basal cells, proliferating NKT cells (NKT-p), IFNG-responsive cells (IFNRep), regulatory T cell (Treg), neutrophils (Neu), monocyte-derived dendritic cells (moDC), nonresident macrophages (nrMa), resident macrophages (rMa), and ionocytes.

**FIGURE 4:**
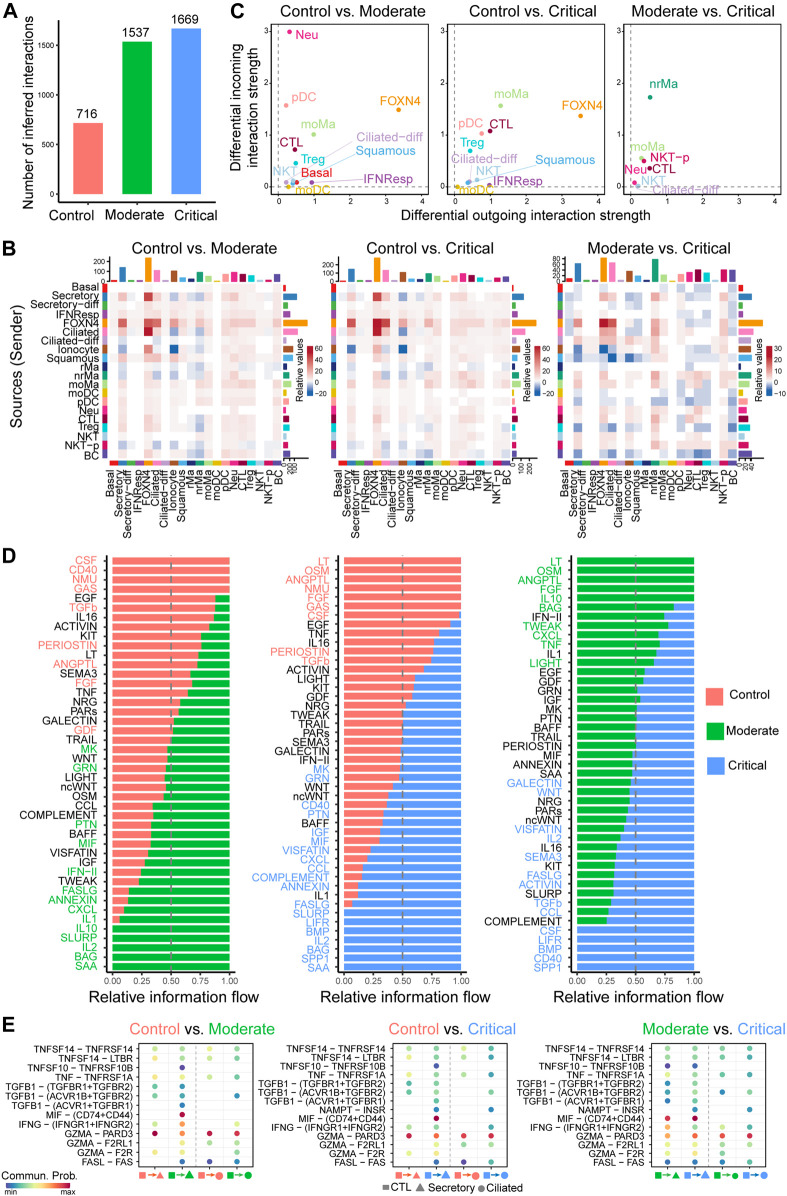
Comparison analysis of cell–cell communication identifies major signaling changes in patients with COVID-19 across control, moderate, and critical cases. **(A)** Comparison of the number of interactions among different cell populations. **(B)** Differential number of interactions in the cell–cell communication network between control, moderate, and critical in greater details. **(C)** Signaling changes of the major cell groups that send or receive signals. Positive values in the differential outgoing (or incoming) interaction strength suggest the increased likelihood being sender (or receiver) in the second dataset compared to the first dataset. **(D)** The comparison of the signaling pathway based on the relative information flow between pairwise datasets. **(E)** Identifying altered ligand–receptor pairs from CTL to secretory and ciliated cells by comparing their communication probabilities between control, moderate, and critical.

After applying our generalized CellChat to the control, moderate, and critical samples separately, we calculated the total numbers of inferred interactions and observed an increased trend as the severity of the disease increases, with the highest interaction number of interactions detected in critical samples (Figure 4A). In more detail, we computed the differential number of interactions for both outgoing and incoming signaling of pairwise cell groups between different severities. Overall, the number of interactions was largely increased in moderate and critical samples compared to control, but exhibited dynamic changes when comparing moderate and critical (Figure 4B). Compared to control, the number of outgoing and incoming interactions of FOXN4, ciliated, and secretory cells in moderate and critical cases is higher. Compared to moderate cases, the outgoing signaling of FOXN4, ciliated, and some immune cells such as nrMa, moMa, Neu, CTL, NKT, and NKT-P was predominantly increased in critical cases (Figure 4B). Next, we examined the major source and target changes in different stages of COVID-19 by computing the differential outgoing and incoming differential interaction strength associated with each cell type (Figure 4C). Interestingly, compared to control, all cell types exhibited increased signaling in either outgoing or incoming signaling. In particular, FOXN4, CTL, moMa, pDC, and Treg in moderate and critical predominantly increased their outgoing and incoming interaction strength. CTL-, nrMa-, moMa-, Neu-, and NKT-associated signaling were further enhanced in critical compared to moderate.

We further focused on the specific signaling changes of two epithelial cell types: secretory and ciliated. Compared to control, certain chemokine and cytokine signaling pathways in moderate and critical were increased in their interaction strength (Supplementary Figure S3A). For the secretory-related signaling, CXCL, IFN-II, and IL1 increased either outgoing or incoming signaling; for the ciliated-related signaling, CCL, IFN-II, and IL2 increased either outgoing or incoming signaling. In addition, we compared the information flow (i.e., the sum of communication probabilities among all pairs of cell populations in the inferred network) for each signaling pathway between control, moderate, and critical samples (Figure 4D). We found that, compared to control, about half of the signaling pathways were highly enriched in moderate and critical (green and blue colors in left and middle panels in Figure 4D). These included many inflammatory signaling pathways such as IFN- II, CCL, CXCL, IL1, and IL2, suggesting that moderate and critical COVID-19 strongly trigger a series of inflammatory responses. Interestingly, compared to moderate, certain inflammatory response–related signaling were diminished in critical, such as OSM, IL10, TWEAK, CXCL, and LIGHT, while other inflammatory response–related signaling were enhanced in critical, such as IL2, IL16, CCL, LIFR, and CD40, suggesting that different inflammatory signaling likely play distinct roles in moderate vs. critical COVID-19.

Given the predominant signaling change of the immune cell CTL and epithelial cell secretory and ciliated, we investigate important ligand–receptor pairs sending from CTL cells to secretory and ciliated cells in moderate and critical. Compared to control, we observed that IFNG-(IFNGR1+IFNGR2) signaling was increased in both moderate and critical and TGFb-related signaling such as TGFB1-(ACVR1B + TGFBR2) was increased in critical compared to moderate (Figure 4E), suggesting the important role of IFN-II signaling in the interplay between immune cells and epithelial cells. Taken together, our comparison analysis revealed crucial signaling changes related to immune and epithelial cells and highlighted the ligand IFNG and its receptors IFNGR1 and IFNGR2 as critical enhanced signaling from CTL to secretory and ciliated cells, which might be responsible for disease severity related to COVID-19.

### Multiscale Signaling Network Elaborates the Signaling Mechanisms of How SARS-CoV-2 Receptor ACE2 is Activated in Epithelial Lung Cells of Severe COVID-19

The binding of virus to the host receptor ACE2 greatly facilitates the infection of the mucosa of the upper respiratory by SARS-CoV-2. Therefore, the understanding of how ACE2 is activated in epithelial lung cells in patients with COVID-19 is crucial for therapeutic intervention of viral infection. To understand the role of cell–cell communication in activating ACE2 expression in the target cell, we constructed a multiscale signaling network by integrating the intercellular communications with the intracellular downstream signaling response (MATERIALS AND METHODS).

**FIGURE 5:**
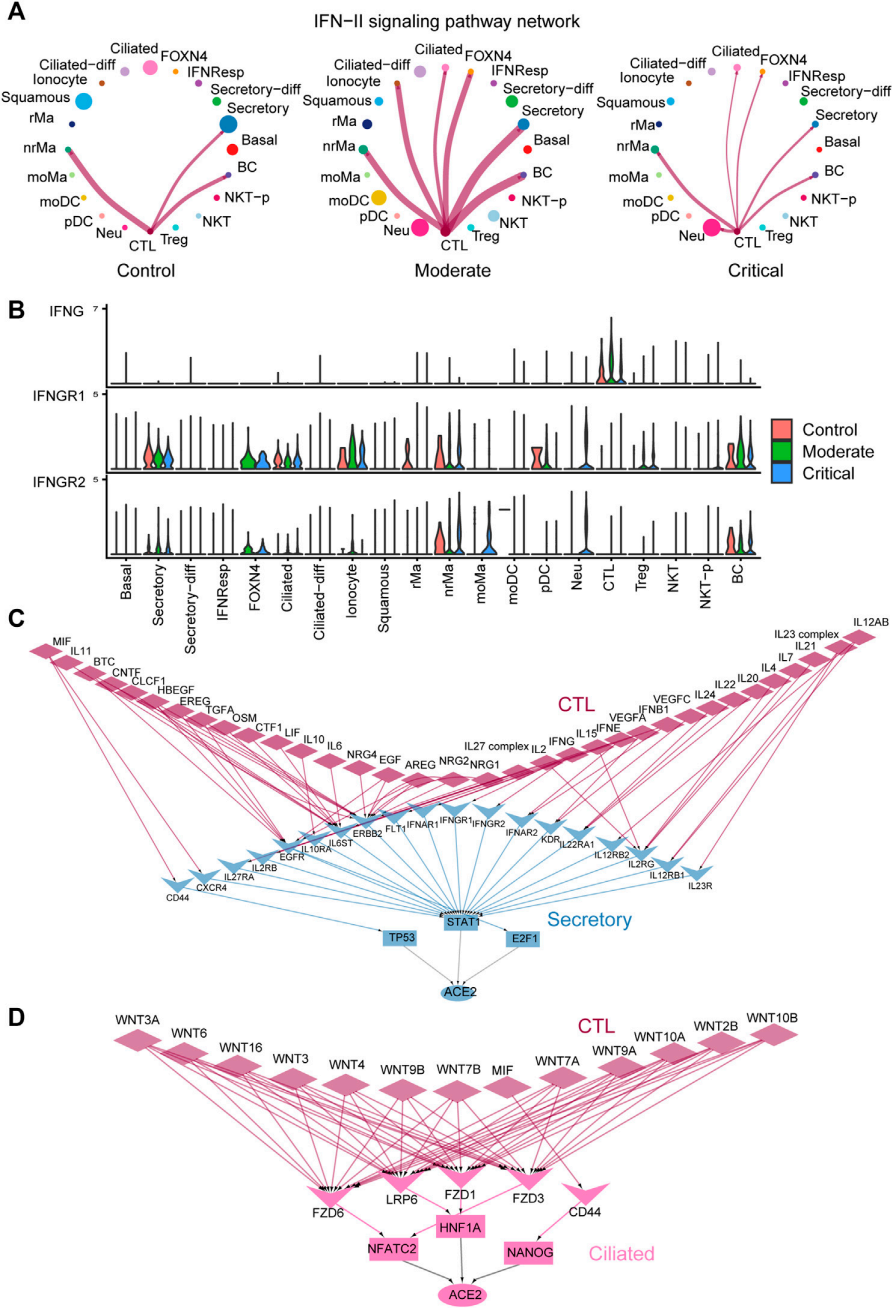
Multiscale signaling network of CTL-to- secretory and CTL-to-ciliated reveals how intercellular communication activates the ACE2 expression *via* TFs in COVID-19. **(A)** Circle plots depicting the inferred IFN-II cell–cell communication networks between different cell groups in control, moderate, and critical. **(B)** Expression of IFN-II signaling–related genes such as IFNG, IFNGR1, and IFNGR2 in control, moderate, and critical COVID-19. **(C)** The inferred multiscale signaling network of CTL-to- secretory in critical COVID-19. **(D)** The inferred multiscale signaling network of CTL-to- ciliated in critical COVID-19.

Our comparison analysis of cell–cell communication among control, moderate, and critical pinpoints the strong activation of cell–cell communication from the immune CTL cells to the epithelial secretory and ciliated cells mediated by IFN-II signaling in the moderate and critical compared to control (Figure 4E). By examining the inferred cell–cell communication network of the IFN-II signaling pathway (Figure 5A), we found that, compared to control, IFN-II signaling is strongly activated in moderate with a stronger interaction strength and more signaling targets. CTL is the dominant signaling source and FOXN4, ciliated, and ionocyte cells emerge as new signaling targets in moderate and critical (Figure 5A). Interestingly, the cell–cell communication strength is slightly diminished in critical compared to moderate, possibly due to the relatively lower expression of the IFNG’s receptors IFNGR1 and IFNGR2 in critical compared to moderate (Figure 5B).

Furthermore, we applied our computational framework of multiscale signaling network construction to study how CTL activates the ACE2 expression in the secretory and ciliated cells through the cell–cell communication. Therefore, we integrated the cell–cell communication network of CTL-to- secretory and CTL-to-ciliated with the downstream signaling network in secretory and ciliated cells, respectively. The downstream signaling network was constructed by integrating the receptor-TFs and TFs-target gene interactions (MATERIALS AND METHODS). Finally, we constructed two multiscale signaling networks for CTL-to- secretory and CTL-to-ciliated, respectively (Figures 5C,D). For the inferred multiscale signaling network of CTL-to- secretory, we observed many interferon, cytokines, and growth factors–related upstream ligands, such as IFNG, IFNB1, IFNE, IL6, IL10, IL2, IL17, OSM, IL4, IL7, VEGFA, EREG, TGFA, and EGF, as well as their corresponding receptors activated in secretory cells, such as IFNGR1, IFNGR2, IL6ST, IL2RG, KDR, and EGFR. Interestingly, our results showed that these ligand–receptor pairs activated three TFs, including STAT1 as the major activator and E2F1 and TP53 as the minor activators, and ACE2 can be activated by these three TFs. These results suggested that STAT1 was the major regulator to activate ACE2 in secretory cells, which is consistent with the previous finding (Chua, et al., 2020) and the known important role of the JAK-STAT signaling pathway during viral infection. Surprisingly, the inferred multiscale signaling network of CTL-to-ciliated showed that WNT signaling was highly activated in ciliated cells, which triggers the activation of three TFs including NFATC2, HNF1A, and NANOG and further activates the downstream target gene ACE2 expression (Figure 5D). Interestingly, previous studies showed that HNF1A is a master regulator of ACE2, and overexpression of HNF1A and ACE2 indicates greater risk of death or cardiovascular disease events (Narula, et al., 2020). In addition, NFATC2 is the predominant NFAT family members in the peripheral immune system and may be as a potential marker related to lung damage (Maremanda, et al., 2020). These results suggest the potential role of these TFs in regulating ACE2 expression in ciliated cells and might be considered as new therapeutic targets. Taken together, our multiscale signaling framework helps to elaborate the signaling mechanisms of how the SARS-CoV-2 receptor ACE2 is activated by TFs in epithelial lung cells of severe COVID-19.

### Comparison CellChat With Other Cell-Cell Communication Tools

The characteristics of CellChat and its comparison with other tools, including iTalk, Connecctome, and NicheNet, are summarized in Supplementary Figure S4A. Briefly, compared to these three tools, the updated CellChat is the only easy-to-use tool that can seamlessly identify signaling changes across any number of scRNA-seq datasets. NicheNet does not perform comparison analysis across distinct datasets. These three tools do not consider the multisubunit structure of ligand–receptor complexes and membrane-bound stimulatory and inhibitory cofactors, which are necessary for certain ligand–receptor binding. Moreover, iTalk and Connectome do not infer the intracellular signaling network.

Since our previous study has already performed comparison analysis with iTalk (Jin et al., 2021) and NicheNet does not explicitly infer the cell–cell communication network, here we only compare CellChat with Connectome in their ability of identifying signaling changes across conditions. We aimed to identify signaling changes responsible for disease severity related to COVID-19. We found that CellChat produced upregulated and downregulated signaling genes that were more differentially expressed compared to Connectome, as reflected by a higher avg [log2(FC)] and −log10 (p_val_adj) of genes in the predicted ligand-receptor pairs (Supplementary Figure S4B). This result suggests that CellChat inferred more significant ligand–receptor interactions that were changed across conditions. By examining the list of inferred signaling pathways, interestingly, Connectome did not produce the IFN-II signaling while CellChat did. This signaling pathway has been shown to be strongly activated in moderate and critical compared to control during COVID infection (Chua et al. 2020). This result indicates CellChat’s ability in predicting dysfunctional signaling pathways across conditions.

## Materials and Methods

CellChat requires gene expression data of cells as the user inputs and models the probability of cell–cell communication by integrating gene expression with prior knowledge of the interactions between signaling ligands, receptors, and their cofactors. Upon inferring the intercellular communication network, CellChat provides functionality for further data exploration, analysis, and visualization (Jin, et al., 2021). Compared to the original CellChat, here we made two important additions. First, the updated CellChat enables systematical comparison analysis of intercellular communication between interacting cells across any number of scRNA-seq datasets rather than limiting to two datasets. In this way, significant signaling changes across multiple conditions or time points can be presented in an intuitive way. Second, the updated CellChat is able to infer the multiscale signaling network linking intercellular communication with intracellular downstream signaling, which helps to better understand how the upstream of the signaling pathway in intercellular communication affects intracellular TFs to regulate the target gene expression.

### Comparison Analysis of Intercellular Communication Between Interacting Cells Across Multiple Datasets

We generalized some functions and analysis in our previously developed R package CellChat, which can then be used for comparative analysis across multiple datasets. Here, we briefly described several key functionalities in the updated CellChat R package.

#### Identification of Important Signaling Sources and Targets in the Intercellular Communication Networks

We identified the dominant signaling sources and targets by defining the outgoing and incoming interaction strength as the out-degree and in-degree centrality metrics in the weighted cellular communication network, where the edge weights are assigned by the communication probabilities computed from CellChat (Jin, et al., 2021). The in-degree refers to the sum of the communication probabilities of incoming signaling to a cell group, while the out-degree is computed as the sum of communication probabilities of the outgoing signaling from a cell group. In this way, we can study the detailed changes in the outgoing and incoming signaling across all significant pathways.

#### Identification of Altered Signaling Pathways by Comparing the Information Flow of Each Signaling Pathway

The information flow for each signaling pathway is defined by the sum of communication probabilities among all pairs of cell groups in the inferred network (that is, the total weights in the network). We can compare the total information flow in the cell–cell communication network of each signaling pathway across different datasets under different conditions, leading to the identification of changes in important signaling pathways.

#### Identification of Signaling Networks With Architecture Difference Across Multiple Datasets Based on Their Network Similarity

CellChat quantifies the similarity of multiple cellular communication networks using structural similarity and functional similarity and performs joint manifold learning and classification of the inferred communication networks based on the computed similarity to identify signaling networks with a certain difference. Here, we focus on the functional similarity, which is calculated by using Jaccard similarity on the basis of the overlap of the major targets and sources in communications defined by:

$$S=\frac{E\left(G\right)\cap E\left(G\prime \right)}{E\left(G\right)\cup E\left(G\prime \right)-E\left(G\right)\cap E\left(G^{\wedge }\prime \right)},$$ (1)

where G  and  G′  are two signaling networks, and E(G) is the set of communications in signaling network G . The higher the functional similarity, the more similar the major senders and receivers are, which means that the two signaling pathways or two ligand–receptor pairs exhibit more similarity. Therefore, two cell-cell communication networks showing less functional similarity suggest that they change their signaling sources and targets across different datasets, implying the difference in network architecture.

### Inference of Multiscale Signaling Network by Integrating Intercellular Communication with Intracellular Signaling Network

The construction of a multiscale signaling network includes the following five steps.

Step 1: Construction of the ligand–receptor subnetwork.

A very important way of information transmission between cells is the interaction between ligands and receptors on the cell surface. The ligand–receptor subnetwork is obtained by applying CellChat to the scRNA-seq data, which infers the biologically significant cell–cell communication network mediated by ligand–receptor interactions based on the database CellChatDB of ligand-receptor pairs in human and mice (Jin, et al., 2021).

Step 2: Construction of the receptor-TF subnetwork.

From the public databases, we get the receptor-TF prior network from the OmniPath database (Türei, et al., 2016; Türei et al., 2021), “kinaseextra” and “pathwayextra” using OmnipathR package (https://github.com/saezlab/OmnipathR).

Step 3: Construction of the TF-target gene subnetwork.

We focused on the cell type–specific signaling network and thus first identified enriched genes and TFs in each cell group. The nonparametric Wilcoxon rank sum test in Seurat v.3 (FindAllMarkers function) was used to perform differential gene expression analysis (min.pct = 0.25, logfc. threshold = 0.25). Genes were considered as enriched genes with an adjusted *p*-value < 0.05. To better model the relationship between TFs and their target genes, we estimated TF activity based on the target’s mRNA expression level from scRNA-seq data using DoRothEA (Garcia-Alonso, et al., 2019) since TF activity is difficult to measure directly and it may be possible to infer changes in the TF activity level from changes in the expression levels of the TF’s target genes. We then identified the enriched TFs in certain cell groups using the differential expression analysis based on the computed TF activity data.

To better infer the TF-target gene regulatory network, we integrated TF-target gene interactions from public databases with scRNA-seq data. We selected TF-target gene interactions with high confidence levels A, B, and C from the OmniPath database. Then, the inference of the TF-target gene regulatory network can be formulated as the following mathematical optimization problem

$$\;\underset{X}{\min}\frac{1}{2}∥A-XB{∥}_{F}^{2}+\frac{1}{2}\lambda_{1}∥X\circ N{∥}_{F}^{2}+\lambda_{2}\overset{m}{\underset{i=1}{\sum }}∥X_{i,:}{∥}_{1},$$ (2)

where X is the TF-target regulatory network we need to infer. A is the target gene expression matrix (rows are target genes and columns are cells. B is the TF activity matrix (rows are TFs and columns are cells). N is the prior TF-target network from the public database (rows are target genes and columns are TFs). The value of each element $N_{ij}$ is 0 or 1, where 0 means that there is no priori connecting edge between $TF_{j}$ and $Target\;gene_{i}$ , and 1 indicates that there is a prior connecting edge. ∘ represents dot product. The last term constrains the sum of the absolute value of all link’s weight coefficients, which can reduce the complexity of the model and make the network sparse, leading to more biologically explanatory results. Here, we choose the two regularization parameters $\lambda_{1}$ and $\lambda_{2}$ as 50 and 10, respectively.

We used the ADMM algorithm to efficiently solve this optimization problem. We rewrite the optimization problem as:

$$\;\underset{X,Z}{\min}\frac{1}{2}∥A-XB{∥}_{F}^{2}+\frac{1}{2}\lambda_{1}∥Z\circ N{∥}_{F}^{2}+\lambda_{2}\overset{m}{\underset{i=1}{\sum ∥}}Z_{i,.}{∥}_{1}.$$ (3)

*Subject to* X−Z=0

The augmented Lagrangian with penalty parameter t > 0 is:

$$L_{t}\left(\text{X},\text{Z},\text{Y}\right)=\frac{1}{2}∥A-XB{∥}_{F}^{2}+\frac{1}{2}\lambda_{1}∥Z\circ N{∥}_{F}^{2}+\lambda_{2}\overset{m}{\underset{i=1}{\sum }}∥Z_{i,.}{∥}_{1}+tY^{T}\left(X-Z\right)+\frac{t}{2}.$$ (4)

We then solved this optimization problem by following the update rules and stop criterion.

Update rules:

1) Update X:

$$X_{k}=arg\underset{X}{\text{min}}L_{t}\left(\text{X},Z_{k-1},{\text{Y}}_{k-1}\right)$$ (5)

2) Update Z:

$$Z_{k}=arg\underset{Z}{\text{min}}L_{t}\left(X_{k},Z,{\text{Y}}_{k-1}\right)$$ (6)

3) Update Y:

$$Y_{k}=Y_{k-1}+t\left(X_{k}-Z_{k}\;\right)$$ (7)

Stop criterion:

dual residual: $S_{k}=-t\left(Z_{k}-Z_{k-1}\right)$

primal residual: $\;R_{k}=X_{k}-Z_{k}$

iteration stops when both ${\left|\left|R_{k}\right|\right|}_{F}$ and ${\left|\left|S_{k}\right|\right|}_{F}$ values become smaller than $\epsilon^{pri}$ and $\epsilon^{dual}$ , respectively,

$${\left|\left|R_{k}\right|\right|}_{F}<\epsilon^{pri},$$

$${\left|\left|S_{k}\right|\right|}_{F}<\epsilon^{dual},$$

where

$$\epsilon^{pri}=\sqrt{n}\epsilon^{abs}+\epsilon^{rel}\max\left\{{\left|\left|X_{k}\right|\right|}_{F},{\left|\left|-Z_{k}\right|\right|}_{F}\right\},$$

$$\epsilon^{dual}=\sqrt{m}\epsilon^{abs}+\epsilon^{rel}{\left|\left|Y_{k}\right|\right|}_{F}.$$

After obtaining the solution X , we determined the weight of the network by considering another proportionality-based association measure “propr” (Quinn, et al., 2017), which was shown to perform very well in inferring gene networks across multiple scRNA-seq datasets and technologies (Skinnider, et al., 2019). We then defined the weights in the TF-target gene network as

$$X_{new}=\omega \cdot X_{model}+\left(1-\omega \right)\cdot X_{propr}.$$ (8)

Here, we took the value of ω as 0.7. The weighted average is an ensemble strategy that has been widely used in many other studies. We also performed comparison analysis of networks inferred using weighted average $X_{new}$ and using $X_{\text{model}}$ . By using a prior network from public databases as a reference, we computed true positive rate (TPR), false positive rate (FPR), and the area under the ROC curve (AUC) and showed that the network inferred with the weighted average produces better results than $X_{\text{model}}$ (Supplementary Figure S5B).

Step 4: Integration of intercellular communication network with intracellular signaling network.

We subset the receptor-TF network by only retaining receptors in the intercellular communication network and TFs in the TF-target gene network. Once we constructed the intercellular communication network mediated by ligand–receptor interactions, the receptor-TF network, and TF-target gene network, we integrated them together to obtain a multiscale signaling network, linking the intercellular communication network with intracellular signaling network.

Step 5: Inference of cell type–specific multiscale signaling network.

Finally, we build the cell type–specific multiscale signaling network based on whether the TFs and target genes were enriched in certain cell types based on the differential expression analysis. Of note, we construct the downstream intracellular signaling network for each dataset separately. To visualize the inferred network, we only retained the top 25 edges based on the inferred edge weights.

### Robustness Analysis of Regularization Parameters

Our model is not sensitive to the regularization parameters within certain ranges. To demonstrate this point, we conducted robustness analysis and varied the regularization parameter values within a certain range to explore the robustness of our model. Specifically, we varied the regularization parameters λ_1_ from 30 to 70 with an increment of 10 and λ_2_ from 5 to 15 with an increment of 5, respectively. We then computed the residual value of the model using five-fold cross-validation under each parameter combination. We observed that the residual value exhibited a slight fluctuation (Supplementary Figure S5A), suggesting that our inference is relatively robust.

### Single-Cell RNA-Seq Datasets, Data Preprocessing, and Analysis

#### Mouse Embryonic Skin scRNA-Seq Datasets

Interfollicular epidermis (IFE) covers the surface of the animal body and is a keratinized stratified squamous epithelium. The datasets (GEO accession codes: GSE154579) we used were published from our previous study (Lin, et al., 2020), containing three developmental stages: E14.5, E16.5, and E18.5 (newborn). The IFE cells were classified into six cell states: basal cells (IFE-B.1 and IFE-B.2), transition cells (IFE-T.1 and IFE-T.2), differentiated cells (IFE-D), and terminally differentiated cells (IFE-TD). Normalized data were used for all the analyses.

#### Mouse Spinal Cord Injury Datasets

Spinal cord injury is the most serious complication of spinal cord injury, often leading to severe dysfunction of the limbs below the injured segment and triggers multiple processes. The published spinal cord injury mouse datasets were downloaded from GEO (accession codes: GSE162610) and included a total of 66,176 cells from the uninjured and 1, 3, and 7dpi tissue (Zhu, et al., 2015). The original study classified these cells into 15 distinct cell groups: microglia, astrocytes, monocytes, macrophages, neutrophils, div−myeloid cells, dendritic cells, lymphocytes, oligodendrocytes, OPCs, neurons, fibroblasts, pericytes, ependymal cells, and endothelial cells. Normalized data were used for all the analyses.

#### COVID-19 Datasets

The processed transcriptomic data of 135,600 cells from patients and control patients with no signs of disease with COVID-19 were downloaded from FigShare: https://doi.org/10.6084/m9.figshare.12436517. This dataset includes eight moderate cases, eleven critical cases, and five control cases (According to the World Health Organization (WHO) guidelines, the severity of the disease is classified) (Chua, et al., 2020). In the control, moderate, and critical samples, each contains 2,982, 82,814, and 49,804 cells. We performed downsampling analysis on the moderate and critical cases with a maximum of 20,000 cells to reduce computational cost. This dataset contains 20 cell types, including ciliated-diff cells (differentiating ciliated), secretory-diff cells (differentiating secretory), ciliated cells, FOXN4+ cells, squamous cells, secretory cells, cytotoxic T lymphocytes (CTL), natural killer T cells (NKT), B lymphocytes (BC), plasmacytoid dendritic cells (pDC), monocyte-derived macrophages (moMa), basal cells, proliferating NKT cells (NKT-p), IFNG-responsive cells (IFNRep), regulatory T cell (Treg), neutrophils (Neu), monocyte-derived dendritic cells (moDC), nonresident macrophages (nrMa), resident macrophages (rMa), and ionocytes. To infer the intracellular signaling network in secretory cells, ciliated cells, and CTL, we only used the top 20 marker genes and the top 50 TFs associated with each cell population based on the differential expression analysis. Normalized data were used for all the analyses.

## Discussion

In this study, we generalized our previously developed tool CellChat to perform comparison analysis of cell–cell communication across multiple conditions or time points and established an optimization-based framework to construct a multiscale signaling network linking intercellular communication with intracellular downstream signaling response. This comparative analysis of the interactions between cell types across different biological conditions is essential for a biologically meaningful understanding of the role of cell–cell communication from scRNA-seq data. We demonstrated the effectiveness of our proposed approaches by studying the signaling changes across three mouse embryonic developmental stages, four time points after mouse spinal cord injury, and patients with different COVID-19 severities (i.e., control, moderate, and critical cases).

We found that our predictions can recapitulate known biology to a substantial degree. For example, the prediction of the WNT signaling pathway as the predominant signaling change during mouse embryonic development is in agreement with our previous finding that WNT signaling can inhibit the expansion of epidermal stem cells and the appearance of abnormal stem cell states during epidermal differentiation (Lin, et al., 2020). Our predictions also reveal many signaling changes that recapitulate previous findings or known biology during mouse spinal cord injury, such as the increased myeloid cell–associated interactions at 1dpi and enhanced OSM and SPP1 signaling, suggesting the important signaling mechanisms of fibrosis mediated by myeloid cells during wound healing after spinal cord injury. We found that the IFN-II signaling pathway has changed significantly in the patients of COVID-19 and can activate the master regulator STAT1 to regulate the downstream ACE2 expression in the secretory cells.

Although recent studies have developed different computational methods to investigate cell–cell communication, our study adds important understanding of the cell–cell communication in several aspects. On the one hand, we provide generalized functions in the CellChat R package for comparative analysis of any number of datasets and even for datasets with not exactly the same cell type compositions under different conditions. It compares the number of interactions; it also identifies changes of major sources and targets in cell groups and changes in signaling pathways and ligand–receptor pairs. The advantage is that the single-cell datasets used for comparative analysis can be any number, not just limited to the comparison between two datasets. Furthermore, we defined signaling similarity by computing the Jaccard similarity between the inferred cell–cell communication networks across different datasets. Our current strategy that combines clustering analysis can help to identify signaling networks that show a relatively large difference in network architecture if they are located in different clusters and far away from each other in the low-dimensional space. However, considering more advanced methods such as statistical tests could likely improve such analysis. We also identified significant changes in senders and receivers of each signaling pathway using network centrality measures such as out-degree and in-degree to characterize the outgoing and incoming interaction strength. Finally, we can use various forms of graphics as output to visualize our results, making the results more intuitive.

On the other hand, we proposed a mathematical optimization model that can infer the TF-target gene network by adding priori network information as a penalty term. Previous studies have also focused on the downstream signaling transduction of cell communication, but these methods like NicheNet and scMLnet primarily use prior network information from public databases, lacking the integration of single cell data in a coherent way. In contrary, our work lies in the integration of mathematical optimization models and prior network information based on a data-driven approach. Although previous studies showed that incorporating such prior information as a network constraint can improve the model performance (e.g., Zhang and Zhang, 2020), reconstruction of the TF-target network directly from single-cell data using a more advanced method such as scLink (Li and Li, 2021) will be likely helpful to build a better multiscale signaling network. Furthermore, we extracted the cell type–specific network based on differential expression analysis and integrated with the upstream intercellular communication network to form a multiscale cellular communication network. In this way, the network we build will likely be more precise and more biologically explanatory.

As single-cell multi-omics data is becoming more common (Argelaguet, et al., 2021; Jin, et al., 2020; Zhang and Nie, 2021), the emergence of these data is a challenging opportunity to build a more systematic cellular communication network. In addition, spatial transcriptomics provide additional information on the cell location (Longo, et al., 2021). Integrating spatial location with scRNA-seq data will likely reduce the false positive inference of cell–cell communication.

## Data Availability

The datasets presented in this study can be found in online repositories. The names of the repository/repositories and accession number(s) can be found in the article/Supplementary Material.

## References

[B1] Almet A. A. Cang Z. Jin S. Nie Q. (2021). The Landscape of Cell-Cell Communication Through Single-Cell Transcriptomics. Curr. Opin. Syst. Biol. 26, 12–23. 10.1016/j.coisb.2021.03.007 33969247 PMC8104132

[B2] Argelaguet R. Cuomo A. S. E. Stegle O. Marioni J. C. (2021). Computational Principles and Challenges in Single-Cell Data Integration. Nat. Biotechnol. 39, 1202–1215. 10.1038/s41587-021-00895-7 33941931

[B3] Armingol E. Officer A. Harismendy O. Lewis N. E. (2021). Deciphering Cell-Cell Interactions and Communication From Gene Expression. Nat. Rev. Genet. 22 (2), 71–88. 10.1038/s41576-020-00292-x 33168968 PMC7649713

[B4] Browaeys R. Saelens W. Saeys Y. (2020). NicheNet: Modeling Intercellular Communication by Linking Ligands to Target Genes. Nat. Methods. 17 (2), 159–162. 10.1038/s41592-019-0667-5 31819264

[B5] Cheng J. Zhang J. Wu Z. Sun X. (2021). Corrigendum to: Inferring Microenvironmental Regulation of Gene Expression from Single-Cell RNA Sequencing Data Using scMLnet With an Application to COVID-19. Brief Bioinform. 22 (2), 1511–1512. 10.1093/bib/bbab015 33448287 PMC7929371

[B6] Chua R. L. Lukassen S. Trump S. Hennig B. P. Wendisch D. Pott F. (2020). COVID-19 Severity Correlates with Airway Epithelium-Immune Cell Interactions Identified by Single-Cell Analysis. Nat. Biotechnol. 38 (8), 970–979. 10.1038/s41587-020-0602-4 32591762

[B7] Garcia-Alonso L. Holland C. H. Ibrahim M. M. Turei D. Saez-Rodriguez J. (2019). Benchmark and Integration of Resources for the Estimation of Human Transcription Factor Activities. Genome Res. 29 (8), 1363–1375. 10.1101/gr.240663.118 31340985 PMC6673718

[B8] Hu Y. Peng T. Gao L. Tan K. (2020). CytoTalk: De Novo Construction of Signal Transduction Networks Using Single-Cell RNA-Seq Data. Sci. Adv. 7 (16), eabf1356. 10.1101/2020.03.29.014464 PMC8046375 33853780

[B9] Jin S. Guerrero-Juarez C. F. Zhang L. Chang I. Ramos R. Kuan C.-H. (2021). Inference and Analysis of Cell-Cell Communication Using CellChat. Nat. Commun. 12 (1), 1088. 10.1038/s41467-021-21246-9 33597522 PMC7889871

[B10] Jin S. Zhang L. Nie Q. (2020). scAI: an Unsupervised Approach for the Integrative Analysis of Parallel Single-Cell Transcriptomic and Epigenomic Profiles. Genome Biol. 21 (1), 25. 10.1186/s13059-020-1932-8 32014031 PMC6996200

[B25] Li W. V. Li Y. (2021). scLink: Inferring Sparse Gene Co-expression Networks From Single-Cell Expression Data. Genomics Proteomics Bioinformatics. 10.1016/j.gpb.2020.11.006 PMC8896229 34252628

[B11] Lin Z. Jin S. Chen J. Li Z. Lin Z. Tang L. (2020). Murine Interfollicular Epidermal Differentiation Is Gradualistic With GRHL3 Controlling Progression From Stem to Transition Cell States. Nat. Commun. 11 (1), 5434. 10.1038/s41467-020-19234-6 33116143 PMC7595230

[B12] Longo S. K. Guo M. G. Ji A. L. Khavari P. A. (2021). Integrating Single-Cell and Spatial Transcriptomics to Elucidate Intercellular Tissue Dynamics. Nat. Rev. Genet. 22, 627–644. 10.1038/s41576-021-00370-8 34145435 PMC9888017

[B13] Maremanda K. P. Sundar I. K. Li D. Rahman I. (2020). Age-Dependent Assessment of Genes Involved in Cellular Senescence, Telomere, and Mitochondrial Pathways in Human Lung Tissue of Smokers, COPD, and IPF: Associations With SARS-CoV-2 COVID-19 ACE2-TMPRSS2-Furin-DPP4 Axis. Front. Pharmacol. 11, 584637. 10.3389/fphar.2020.584637 33013423 PMC7510459

[B14] Milich L. M. Choi J. S. Ryan C. Cerqueira S. R. Benavides S. Yahn S. L. (2021). Single-Cell Analysis of the Cellular Heterogeneity and Interactions in the Injured Mouse Spinal Cord. J. Exp. Med. 218 (8), e20210040. 10.1084/jem.20210040 34132743 PMC8212781

[B15] Milich L. M. Ryan C. B. Lee J. K. (2019). The Origin, Fate, and Contribution of Macrophages to Spinal Cord Injury Pathology. Acta Neuropathol. 137 (5), 785–797. 10.1007/s00401-019-01992-3 30929040 PMC6510275

[B16] Narula S. Yusuf S. Chong M. Ramasundarahettige C. Rangarajan S. Bangdiwala S. I. (2020). Plasma ACE2 and Risk of Death or Cardiometabolic Diseases: a Case-Cohort Analysis. The Lancet. 396 (10256), 968–976. 10.1016/s0140-6736(20)31964-4 PMC7529405 33010842

[B17] Plikus M. V. Wang X. Sinha S. Forte E. Thompson S. M. Herzog E. L. (2021). Fibroblasts: Origins, Definitions, and Functions in Health and Disease. Cell. 184 (15), 3852–3872. 10.1016/j.cell.2021.06.024 34297930 PMC8566693

[B18] Quinn T. P. Richardson M. F. Lovell D. Crowley T. M. (2017). Propr: An R-Package for Identifying Proportionally Abundant Features Using Compositional Data Analysis. Sci. Rep. 7 (1), 16252. 10.1038/s41598-017-16520-0 29176663 PMC5701231

[B19] Raredon M. Yang J. Garritano J. Wang M. Niklason L. E. (2021). Connectome: Computation and Visualization of Cell-Cell Signaling Topologies in Single-Cell Systems Data. bioRxiv. 10.1101/2021.01.21.427529 PMC8906120 35264704

[B20] Sha Y. Wang S. Bocci F. Zhou P. Nie Q. (2020). Inference of Intercellular Communications and Multilayer Gene-Regulations of Epithelial-Mesenchymal Transition From Single-Cell Transcriptomic Data. Front. Genet. 11, 604585. 10.3389/fgene.2020.604585 33488673 PMC7820899

[B21] Shao X. Lu X. Liao J. Chen H. Fan X. (2020). New Avenues for Systematically Inferring Cell-Cell Communication: Through Single-Cell Transcriptomics Data. Protein Cell. 11 (12), 866–880. 10.1007/s13238-020-00727-5 32435978 PMC7719148

[B22] Skinnider M. A. Squair J. W. Foster L. J. (2019). Evaluating Measures of Association for Single-Cell Transcriptomics. Nat. Methods. 16 (5), 381–386. 10.1038/s41592-019-0372-4 30962620

[B23] Türei D. Korcsmáros T. Saez-Rodriguez J. (2016). OmniPath: Guidelines and Gateway for Literature-Curated Signaling Pathway Resources. Nat. Methods. 13 (12), 966–967. 10.1038/nmeth.4077 27898060

[B24] Türei D. Valdeolivas A. Gul L. Palacio-Escat N. Klein M. Ivanova O. (2021). Integrated Intra- and Intercellular Signaling Knowledge for Multicellular Omics Analysis. Mol. Syst. Biol. 17 (3), e9923. 10.15252/msb.20209923 33749993 PMC7983032

[B26] Wang Y. Wang R. Zhang S. Song S. Wang L. (2019). iTALK: An R Package to Characterize and Illustrate Intercellular Communication. bioRxiv. 10.1101/507871

[B27] Zhang L. Nie Q. (2021). scMC Learns Biological Variation Through the Alignment of Multiple Single-Cell Genomics Datasets. Genome Biol. 22 (1), 10. 10.1186/s13059-020-02238-2 33397454 PMC7784288

[B28] Zhang L. Zhang S. (2020). A General Joint Matrix Factorization Framework for Data Integration and its Systematic Algorithmic Exploration. IEEE Trans. Fuzzy Syst. 28 (9), 1971–1983. 10.1109/tfuzz.2019.2928518

[B29] Zhu Y. Soderblom C. Krishnan V. Ashbaugh J. Bethea J. R. Lee J. K. (2015). Hematogenous Macrophage Depletion Reduces the Fibrotic Scar and Increases Axonal Growth After Spinal Cord Injury. Neurobiol. Dis. 74, 114–125. 10.1016/j.nbd.2014.10.024 25461258 PMC4323620

