# Supplementary material for: Identification of Intercellular Signaling Changes Across Conditions and Their Influence on Intracellular Signaling Response From Multiple Single-Cell Datasets
Source: Front Genet. 2021 Nov 11;12:751158. doi: 10.3389/fgene.2021.751158 (PMC8632559; doi:10.3389/fgene.2021.751158)
Supplement: Supplementary file 1 [file Presentation1.pdf]

SUPPLEMENTARY MATERIAL for

Identification of Intercellular Signaling Changes across Conditions and Their Influence on Intracellular Signaling Response From Multiple Single-Cell Datasets

Mengqian Hao, Xiufen Zou and Suoqin Jin

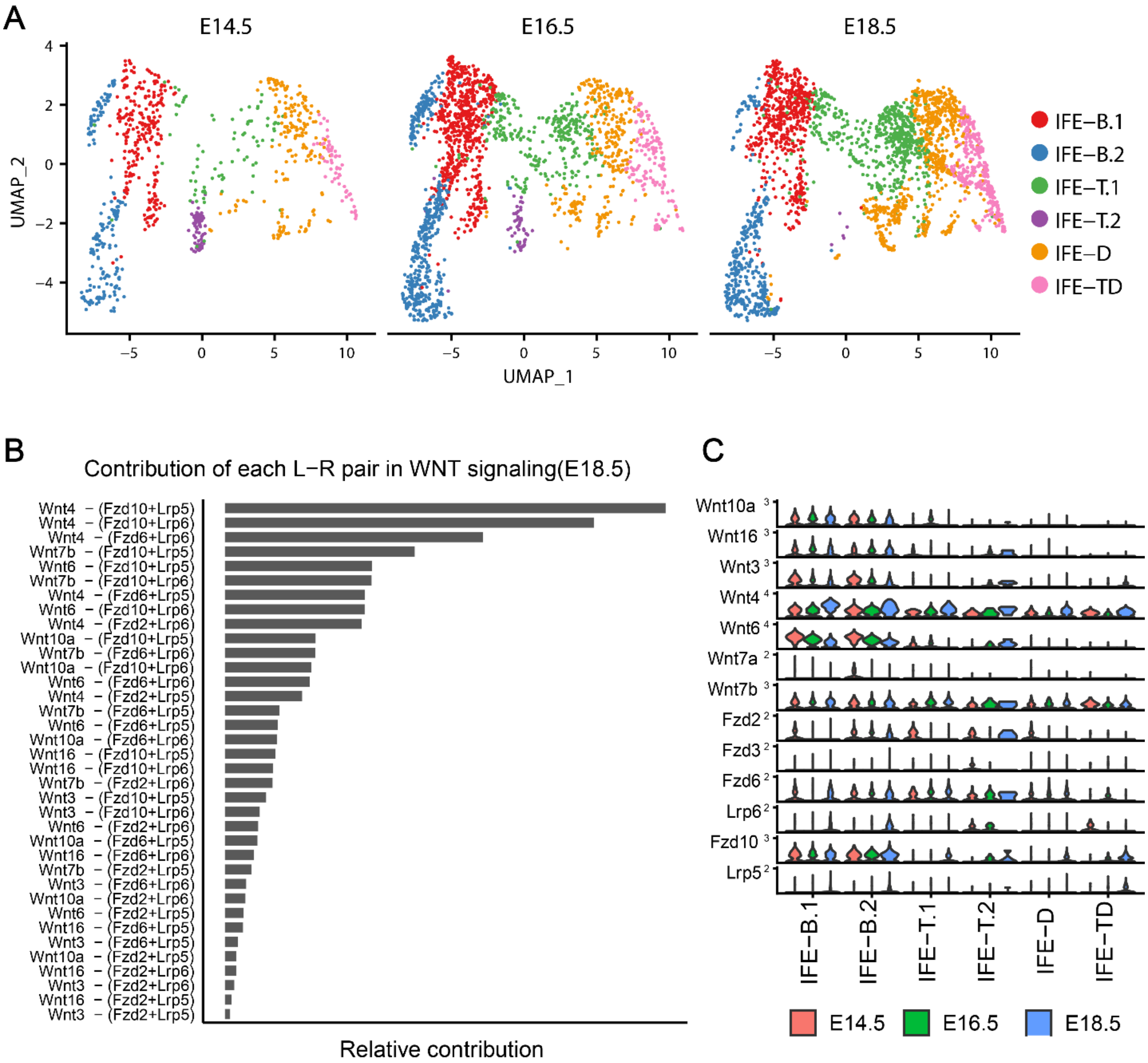

Supplementary Fig. 1 | (A) UMAP plot of cells in each developmental stage. Cells are colored by the cell identity. **(B)** The relative contribution of each ligand-receptor pair to the overall WNT signaling pathway. **(C)** The gene expression distribution of signaling genes related to L-R pairs in WNT signaling pathway in different types of cells between different stages during mouse embryo development.

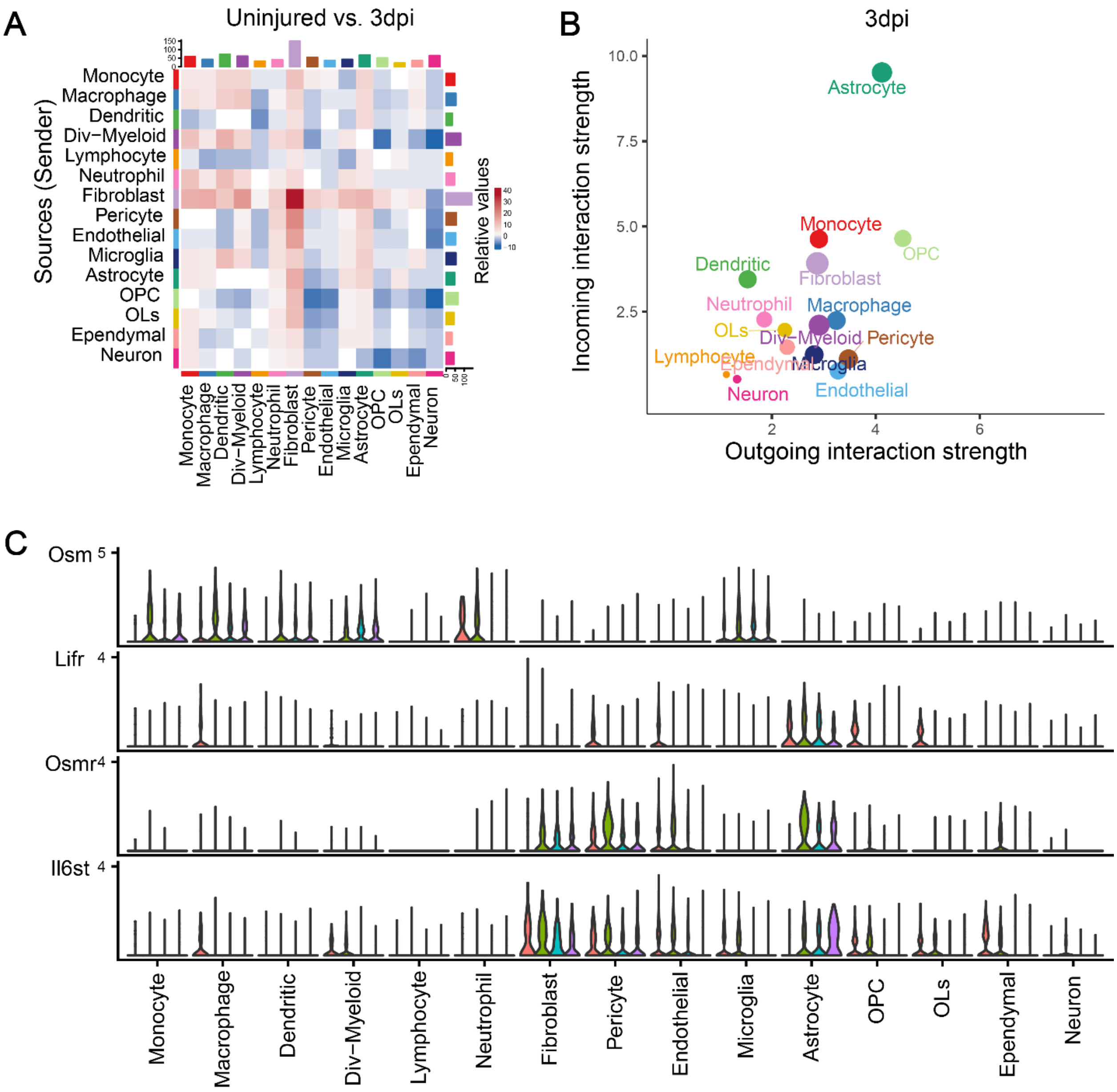

Supplementary Fig. 2 | (A) Differential number of interactions between uninjured and 3dpi. **(B)** Scatter plot showing the outgoing and incoming interaction strength of each cell population in 2D space at 3dpi. **(C)** The gene expression distribution of signaling genes related to L-R pairs in OSM signaling pathway in 15 cell groups at uninjured (red), 1 (green), 3 (blue), and 7dpi (purple).

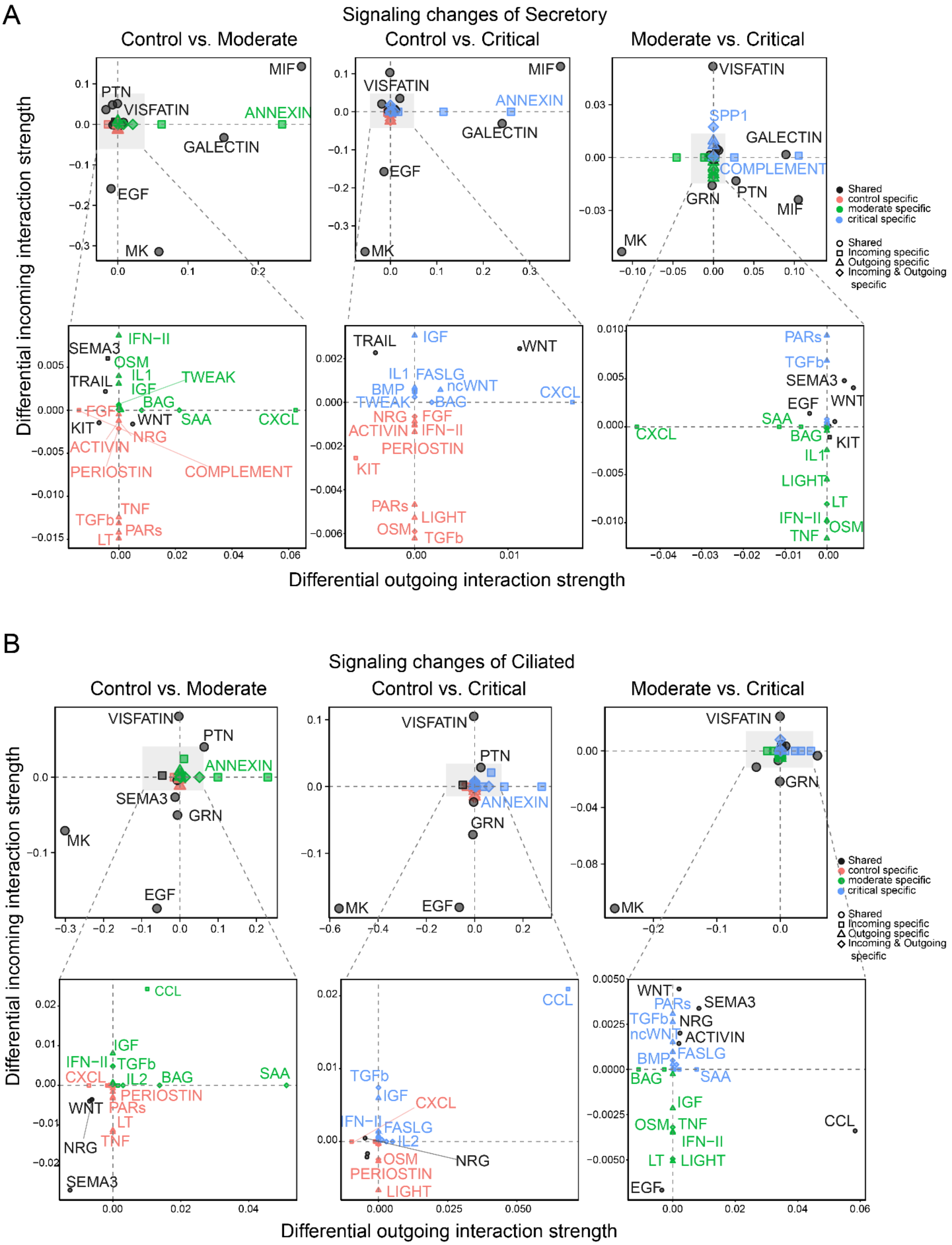
A
Signaling changes of Secretory
Control vs. Moderate
Control vs. Critical
Moderate vs. Critical
MIF
PTN
VISFATIN
ANNEXIN
GALECTIN
EGF
MK
SPP1
COMPLEMENT
GRN
Shared
control specific
moderate specific
critical specific
Shared
Incoming specific
Outgoing specific
Incoming & Outgoing specific
IFN−II
SEMA3
OSM
TRAIL
IL1
IGF
TWEAK
BAG
FGF
KIT
WNT
SAA
CXCL
NRG
ACTIVIN
PERIOSTIN
TNF
TGFb
PARs
LT
FASLG
BMP
ncWNT
LIGHT
Differential incoming interaction strength
Differential outgoing interaction strength
B
Signaling changes of Ciliated
Control vs. Moderate
Control vs. Critical
Moderate vs. Critical
VISFATIN
PTN
ANNEXIN
SEMA3
GRN
MK
EGF
Shared
control specific
moderate specific
critical specific
Shared
Incoming specific
Outgoing specific
Incoming & Outgoing specific
CCL
IGF
IFN−II
TGFb
CXCL
IL2
BAG
SAA
PERIOSTIN
WNT
PARs
LT
NRG
TNF
IL-2
FASLG
OSM
LIGHT
ACTIVIN
ncWNT
BMP
Differential incoming interaction strength
Differential outgoing interaction strength

Supplementary Fig. 3 | (A) Secretory associated signaling changes across control, moderate and critical. **(B)** Ciliated associated signaling changes across control, moderate and critical.

A

Tool	Method Overview	Visualization	The Number of Datasets Can Be Compared and Analyzed	Cell-Cell Communication Score	Consideration of Subunit Structure	Code Availability
CellChat	●Comparison analysis of intercellular communication between interacting cells across multiple datasets<br>●Identify signaling changes at cell populations, signaling pathway and ligand-receptor pairs	Heatmap, Chord diagram, Bubble plot, Bar plot, Scatter plot, Hierarchy plot, Circle plot, Dot plot, River plot	Any	Mass-action-based Probability	Y	https://github.com/sqjin/CellChat
Connectome	●Explore cell-cell connectivity patterns based on ligand/receptor data<br>●Compare cell-cell signaling across two or more tissue systems by comparing sending- and receiving- centrality	Chord diagram, EdgeDot plot	2（Note: CompareCentrality allows an input of any number of connectomes）	Expression Product	N	https://github.com/msraredon/Connectome/
iTALK	●Identify significant changes of interactions between different clusters by differentially analysis	Chord diagram	2	Differential Combinations	N	https://github.com/Coolgenome/iTALK
NicheNet	●Infer active ligand-target links between interacting cells by using prior information on potential ligand-target links<br>●Measure the predictive activity of the ligand and affected targets by ligand-target regulatory potential scores	Heatmap,Chord diagram, Dot plot	1	Personalized-Page Rank-based Score	N	https://github.com/saeyslab/nichenetr

B

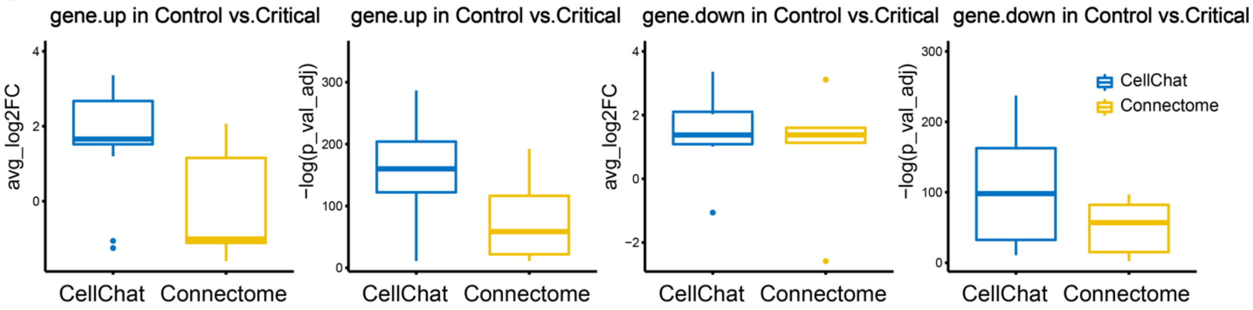

Supplementary Fig. 4 | (A) Characteristics of CellChat and other relevant tools. **(B)** Comparison of the performance of CellChat with Connectome on the COVID-19 scRNA-seq dataset.

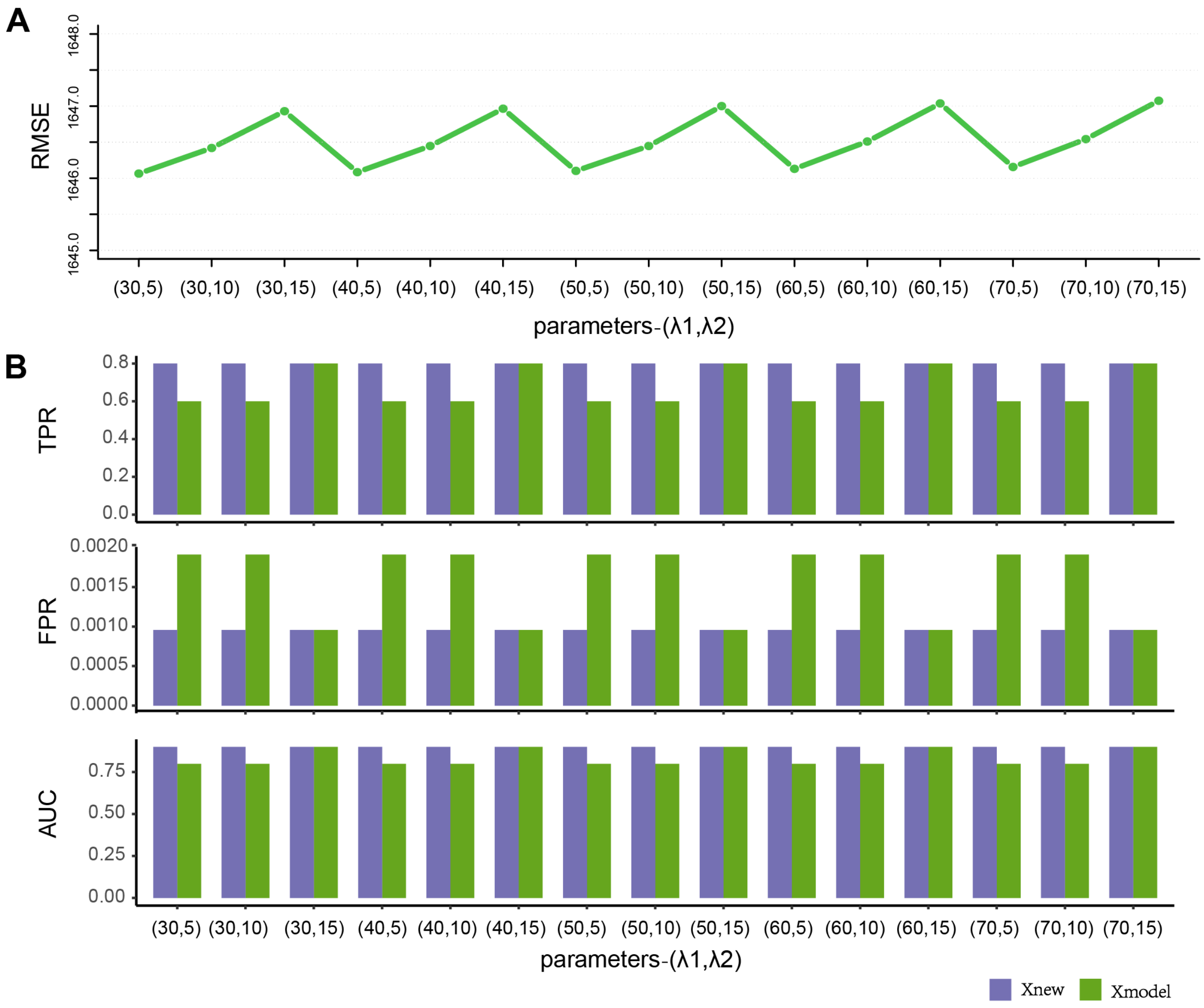

Supplementary Fig. 5 | (A) The root-mean-square error (RMSE) of the model using five-fold cross-validation with the two regularization parameters λ_1 and λ_2 in the range of (30, 70) and (5, 15), respectively. **(B)** Comparison of the calculated TPR, FPR and AUC when the network is inferred with (X_{new}) or without weighted average (X_{model}).
